# The sternum reconstruction: Present and future perspectives

**DOI:** 10.3389/fonc.2022.975603

**Published:** 2022-10-28

**Authors:** Beatrice Aramini, Valentina Masciale, Lorenzo Federico Zini Radaelli, Rossella Sgarzani, Massimo Dominici, Franco Stella

**Affiliations:** ^1^ Division of Thoracic Surgery, Department of Experimental, Diagnostic and Specialty Medicine—DIMES of the Alma Mater Studiorum, University of Bologna, G.B. Morgagni—L. Pierantoni Hospital, Forlì, Italy; ^2^ Cell Therapy Laboratory, Department of Medical and Surgical Sciences, University of Modena and Reggio Emilia, Modena, Italy; ^3^ Center of Major Burns, Plastic Surgery Unit, Maurizio Bufalini Hospital, Cesena, Italy; ^4^ Division of Oncology, Department of Medical and Surgical Sciences, University of Modena and Reggio Emilia, Modena, Italy

**Keywords:** sternum reconstruction, 3D printing, mesenchymal stem cells, sternum allograft, sternal tumors sternum, reconstruction, Prothesis, 3D materials

## Abstract

Sternectomy is a procedure mainly used for removing tumor masses infiltrating the sternum or treating infections. Moreover, the removal of the sternum involves the additional challenge of performing a functional reconstruction. Fortunately, various approaches have been proposed for improving the operation and outcome of reconstruction, including allograft transplantation, using novel materials, and developing innovative surgical approaches, which promise to enhance the quality of life for the patient. This review will highlight the surgical approaches to sternum reconstruction and the new perspectives in the current literature.

## Introduction

Chest wall corrections are generally concerned with the resection of primary locally invasive chest wall malignancies or metastatic tumors (1, 2). The correct approach for the reconstruction depends on the size, location, and depth of the tumor, as well as the vitality of the surrounding tissues. The aim is to obtain clean surgical margins to afford the patients the longest time survival while avoiding recurrence. The majority of surgeons consider a defect bigger than 5 cm or including more than four ribs as a mandatory case for reconstruction due to the possibility of further complications related to the instability of the chest wall (2, 3). Moreover, certain defects, such as some apicoposterior defects, even of bigger dimensions, may not need reconstruction due to sufficient support provided by the shoulder or by the scapula (2, 3). The first goal of a chest wall reconstruction is to maintain the stability of the thorax, preserving the lung functions and protecting the intrathoracic organs, while minimizing the deformity which may derive from the resection (4, 5). One of the most beneficial approaches in the last few decades is the discussion of each clinical case by a multidisciplinary team including thoracic surgeons, plastic surgeons, neurosurgeons, and radiation oncologists to provide the optimal setting and procedures tailored to the patient (4, 6, 7). The choice of the proper materials for the reconstruction is also necessary to obtain the optimal aesthetic effect and physical comfort (4, 6, 7).

One of the most challenging procedures for thoracic surgeons is the removal of the entire sternum for a tumor or infection infiltrating the bone (8, 9), which is quite frequent in tumors growing in the anterior part of the chest. The greater challenge is the reconstruction, which must guarantee the protection of the underlying visceral components, the space behind the sternum, and restoration of the stability of the chest wall and pulmonary function (9).

The capacity to maintain the stability of the chest wall has been extensively studied for its crucial role in preserving the dynamics of breathing (10, 11). Recently, computer simulations have helped guide reconstruction of the chest and prevent possible functional problems after surgery (12, 13). Reduction of thorax expansion may compromise the volume of the chest, with 20% loss of its normal capacity (6). The type of prosthesis is also critical because most of the patches are non-absorbable and synthetic and the patients are often young, with a long-life expectancy (10–14).

**Figure 1:**
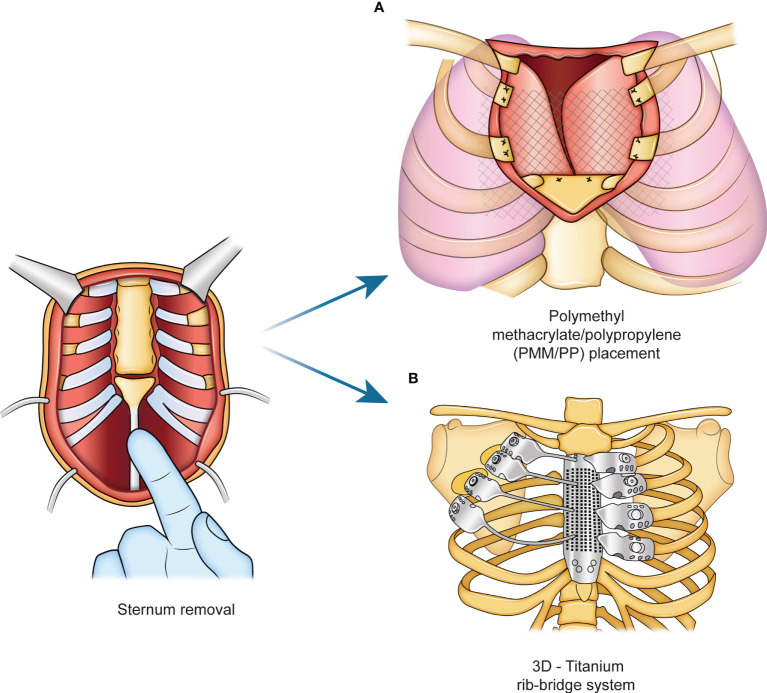
Standard procedure for sternal reconstruction. Current approaches for sternum reconstructive surgery rely on the use of a sandwich implant with a polymethyl methacrylate/polypropylene (PMM/PP) shown in panel **(A)**, and a titanium rib-bridge system, shown in panel **(B)**.

The prosthesis materials are designed to stretch uniformly, inducing a uniform tension at the extremities where they will be fixed (15–17). They are generally well tolerated if covered by viable tissue, although some reports described an infection rate between 10 and 25% for the use of synthetic meshes, which needed to be removed due to infection. Other interesting materials have been developed to avoid this problem, such as vinyl meshes, due to their flexible characteristics and biocompatibility, or the bovine pericardium prosthesis, which is completely biological and mitigates infection or contamination (18). Furthermore, the scientific community is trying to identify the best approaches to cover the chest, especially after sternum removal (11, 16). Currently, sternal reconstruction methods often employ a sandwich approach using a polymethyl methacrylate/polypropylene (PMM/PP) implant and a soft tissue flap (19, 20) (**Figure 1**). Another approach involves the use of a titanium rib-bridge system in addition to soft tissue flaps (21, 22) (**Figure 1**).

Long-term results related to PMM/PP hardware failure have found a solution using the rigid reconstruction of the sternum with a double-barrel free fibula flap plus titanium plates, with the soft tissues of the free flap as coverage (22, 23). This approach provides better stability due to the improved biomechanical design (23–25).

Moreover, sternectomy due to an infection after a cardiothoracic operation has an incidence between 1 and 4% (26); however, reconstruction of the bone provides long-term results, without significant morbidity, although several reports have emphasized the importance of coverage with a visceral component or muscles flaps (27–29). The most important step in sternal reconstruction is the setting of the anterior chest wall to avoid respiratory problems that may arise due to hypomobility of the chest wall (30). In recent decades, different techniques and materials have been used for sternal reconstruction, and currently (31), attention has been given to allograft sternum implantation for better aesthetic results and a more “natural” definition of the anterior chest, without immunosuppression.

## Sternum reconstruction for oncological reasons and infections

Primary malignant sternal tumors (PMSTs) are infrequent tumors, and most of them present at the stage of infiltration of the sternum and soft tissues (32, 33). Radical resection may be the most successful standard treatment, although the local aggressiveness of the tumor makes the surgical approach particularly complex and is associated with a high risk of recurrence (4, 32, 34).

Musculocutaneous flaps have been used to successfully cover extensive skin excisions (4, 28, 29, 35, 36). A representative demonstration of this method was shown in 2004 by Alain R. Chapelier et al., who reported 38 patients undergoing curative resection for PMST (37). The resections included the affected sternum, with partial or complete removal and *en bloc* asportation of the closed area. The sternal defects were reconstructed using a mesh for chest wall stability and the pectoralis major (PM) muscles with skin advancement or latissimus dorsi musculocutaneous flap to reconstruct the soft tissue cover.

The results are generally satisfactory, with low mortality. Furthermore, the stability of the chest can be supported by various prosthetic materials, such as two layers of Marlex mesh (MMM), as proposed by several authors (38–40), or a polytetrafluoroethylene (PTFE) patch (33) (**Figure 1**). Recently, another approach was introduced with methyl methacrylate bars to reduce the amount of prosthetic material, and thus, the risk of infection (7, 41). However, the PM is the most commonly used material for the correction of sternal defects, especially in men, but in women, skin closure or grafting of the donor site is commonly conducted (28, 29). In particular, musculocutaneous flaps guarantee the best aesthetic results and represents a well-vascularized soft tissue cover (28, 29).

Another less frequently used approach is momentum interposition, especially in patients undergoing resection of an irradiated sternum or recurrent tumors, such as sarcomas (42, 43). Although satisfactory results in high-grade tumors have not been reported, in patients with limited local recurrence or one metastasis, resection may be possible with good long-term survival (5, 44–47). Nevertheless, in large sternal defects or patients with an irradiated sternum, reconstruction using musculocutaneous flaps is preferred because of the reduced risk of infection (24, 40, 46). Additionally, the use of methyl methacrylate mesh is recommended for reconstruction after complete sternectomy (48).

Secondary sternal tumors are infrequent and are associated with breast, thyroid, or kidney metastasis (49–51). They represent 15% of all sternal tumors and involve mainly the body of the sternum. Because they are infrequent, the scientific community has not reached a consensus regarding their treatment (50). Chemoradiotherapy and hormonal therapy are generally considered the gold standards of treatment (51, 52).

## Surgical techniques and prosthesis

The most common materials for sternum reconstruction are PTFE and MMM because they provide rigidity of the chest (11, 51). When PTFE is used for extensive anterior chest wall defects requiring rib resection up to the entire lateral aspect of the sternum, the reconstruction is based on a series of sternal punches passing through the sternum to accommodate the anchoring sutures of either the PTFE or the MMM (53).

### The methyl methacrylate mesh

Methyl methacrylate consists of a sandwich of two mesh layers to maintain the rigidity of the reconstruction. This product has been used since the 1980s and for several years it has been considered the best choice for the sternum and the entire or partial chest wall reconstruction (54). It is usually set by the thoracic surgeon with the first layer of polypropylene material fixed on the ribs, and the methyl methacrylate is generally used as a cover for the prosthesis, becoming an integral part of the chest support. This approach is particularly useful for massive chest wall demolitions, especially anteriorly and laterally, to prevent chest deformity (55). On the other hand, the material characteristics of methyl methacrylate do not include fluid permeability, and this could be an unfavorable due to the risk of infection, pain, and rigidity of the thorax (55–57). The most frequent complications regarding the use of methacrylate (11) are fractures and infections, which have been described in 10–20% of patients, followed by the necessity to remove the prosthesis (11, 33). The scientific community and experts in the field agree that coverage with soft tissues is necessary to guarantee satisfactory long-term results (34, 58).

### PTFE

PTFE is another material frequently used for chest wall reconstruction (30). The material is flexible and easily conforms to the chest. The thickness of the mesh provides a permanent tight suture and good chest stability. Similar to MMM, PTFE is useful for large correction of the thorax, and it is recommended to cover it with viable tissue (11). The only difference compared to MMM is that, even if it becomes infected, immediate removal is not suggested, but rather, the scientific literature advises removing it after 6–8 weeks from infection, so the scar tissue can support the chest after the mesh removal (34, 59).

### Titanium plates

In the last few decades, new approaches and materials have been developed for prosthetic surgery, including thoracic surgery. The use of titanium is popular because it exhibits high strength, low weight, and intrinsic diamagnetic characteristics, which permits patients to continue using the magnetic resonance imaging diagnostic tool (60). The most important trait of titanium is its high biocompatibility. Different models have been used recently by surgeons, from the Borrelly steel staple-splint system to STRATOS bars, which are reportedly comfortable in regard to remodeling and fixing on the ribs (61, 62). The improved results are attributed to locking the bars in place using at least three screws to guarantee the stability of the chest in the area where the terminal part of the clean resected ribs margins needs to be fixed (61, 62). Several studies confirmed that the titanium bars may be more beneficial in cases of large thorax reconstruction, not only for guaranteeing the stability of the chest but also for preventing respiratory problems and infections (34). In particular, only a few complications have been observed, such as dislocations or ruptures of the bars, with an incidence frequency of around 0 to 11% of cases (63).

Moreover, titanium plates in association with acellular collagen matrixes or cryopreserved homografts may be an appropriate alternative in cases of re-operation or operation of a highly irradiated area (51, 53, 64). The titanium bars may be formed to the desired length and are anchored to the ribs to prevent fracture or dislocation, which occurred in only one patient who required plate replacement with acellular collagen matrix patching (64). The titanium plates are usually implanted at a 2:1 ratio, depending on the number of ribs resected (64). Moreover, if an acellular collagen matrix prosthesis is selected, a combination of titanium plates and an acellular collagen matrix patch can be used (65, 66). Another approach involves using titanium plates without an internal coverage material (7, 13, 22, 33, 38, 53).

However, the necessity to cover large reconstructions, often considered one of the main causes of high morbidity and mortality, led to the development of more innovative approaches. In particular, the versatility of materials has been considered a point of interest, and for this reason, the introduction of 3D-printed sternum prostheses has introduced a new paradigm of “chest wall reconstruction” (67, 68). A baseline high-resolution computed tomography scan is used to define a 3D model of the thorax and tumor mass using specific software (69). Through the use of powdered titanium and electron-beam melting technology, each layer is constructed and modeled as a personalized sternum to ensure optimal anchorage to the ribs and to ensure clean margins after surgical resection (59). Recently, several authors have reported customizing a titanium sternum model after resection with significant results (70).

Other interesting materials include carbon-fiber molds or alumina-ceramic models, which can be produced in a very short time, usually around 7 days, with very good aesthetic results (71). In particular, long-term results related to this new generation of materials showed that they remained very stable, even years after implantation (71, 72). For both the approaches, regarding the use of new materials and standards, a higher complication rate has been reported in patients with severe co-morbidities and older age (71–73). One equally important aspect is the cost of 3D-printed models, which depends on the size and thickness of the prosthesis. The use of traditional materials (i.e., a combination of titanium bars and mesh) is much cheaper (between 400 and 500 €) than the use of alumina prostheses, which usually cost around 10,000–15,000 € each (74).

## Allogenic sternal allografts and the future of regenerative medicine in sternum reconstruction

**Figure 2:**
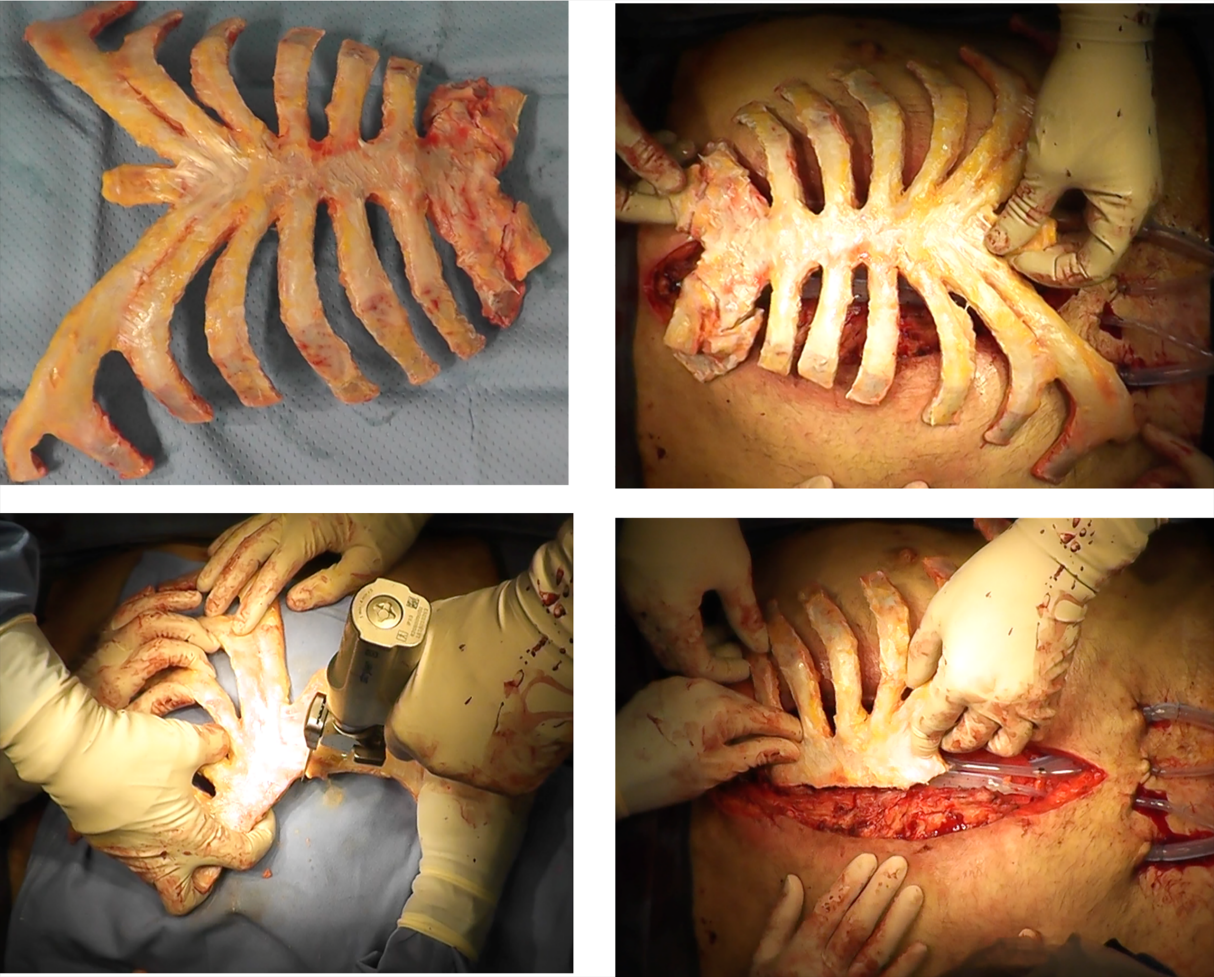
The sterno-chondral graft preparation. The sternum is usually derived from a tissue bank *via* an aseptic procedure.

Cryopreserved allografts and homografts, recovered from cadaveric donors and stored at –80°, have also been considered as a possible solution to reconstruct the thorax after a large chest wall demolition, or in cases of severe local infection (75). These materials may be more useful than prosthetic materials since they can be incorporated into native tissue along with the revascularization and cell populations (76). However, this approach is not widely used due to the challenges associated with identifying a donor in a short time period (77, 78). The sternochondral graft is usually derived from a tissue bank *via* an aseptic procedure, according to Italian rules (**Figure 2**). An antibiotic solution is added for 72 hours at 4°C, and cryopreservation at -80 °C is necessary to preserve the allograft from immunogenic alterations (77, 78). The sternum is then defrosted at 4–6 °C for 12 hours the day before surgery and is placed into a sterile bag. The graft is generally defrosted in a 0.9% NaCl solution with antibiotics (77, 78). The surgical procedure involves the removal of the sternum and associated subcutaneous or cutaneous tissues.

The most common approach to cover the allograft is using a PM muscle-flap reconstruction to ensure an ideal fit with the chest wall of the recipient (21, 28, 29, 40). Titanium bars are also fixed on the sternum to preserve the stability of the anterior chest. The PM muscle flaps are usually used, even in case of a good reconstruction, for an aesthetically favorable result (79).

Despite the availability of common materials used for sternum reconstruction, new regenerative approaches have been explored in recent decades (80). Scientists are trying to identify a strategy to promote tissue regeneration with bone remodeling using cell therapy specifically based on mesenchymal stem cells (MSCs), which appear to play a strategic role in bone healing, to implement sternal nonunion (81). In addition, MSCs have been considered for cartilage restoration after injury. Cartilage can self-repair in a complex structure with low metabolic capacity (82). Surgical approaches that involve the management of cartilage usually include microfractures and autologous osteochondral transplantation; however, there is currently a tendency to prefer those standards of care over the use of regenerative treatments. These treatments should be considered because they could replace surgical procedures that provide only short-term restoration in favor of a long-term regeneration (82, 83).

**Figure 3:**
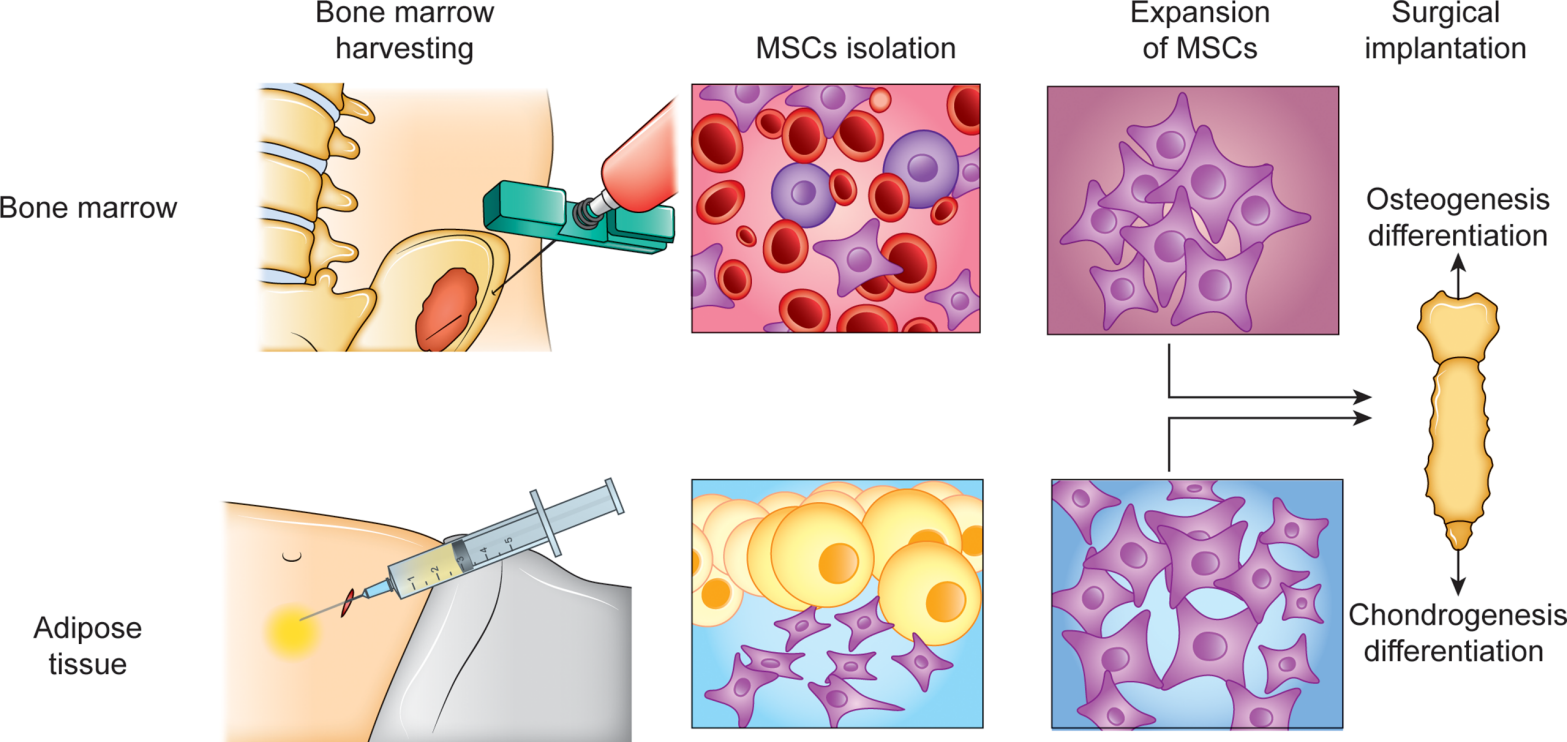
Regenerative approach for sternum reconstruction. Cell therapy approach specifically based on the transplant of MSCs for sterno-chondral reconstruction is a method for long term regeneration. Bone marrow (BM) and adipose tissue (AD) as sources for MSCs isolation, using their capacity to differentiate into both osteogenic and chondrogenic cells.

One of the main advantages of MSCs in the scope of cellular therapies is that MSCs, through a paracrine effect, exhibit anti-inflammatory activity, and thus, reduce fibrosis and anti-apoptotic activity while promoting cell proliferation (59). The optimal source of MSC retrieval is still debated since different tissues have been identified as potential sources of MSCs, such as bone marrow and adipose tissue (84, 85) (**Figure 3**). Bone marrow mesenchymal stem cells (BMSCs) are the most widely used for musculoskeletal regeneration because they can differentiate into adipogenic, osteogenic, chondrogenic, and myogenic cells; however, adipose-derived mesenchymal stem cells (ADSCs) have shown greater genetic stability, proliferation capacity, and less senescence than BMSCs (85, 86) (**Figure 3**).

The new strategies in regenerative medicine involve more complex but tailored approaches in the field of bone and cartilage reconstruction. This is a new chapter in the field of tissue regeneration, although several limitations need to be clarified. In particular, numerous preclinical and clinical trials have confirmed that MSCs can differentiate into cartilage tissue under the influence of chondrogenic factors, facilitating their use for the repair of injured cartilage (87). Moreover, during the process of differentiation, MSCs can produce various extracellular matrices (ECMs) that are essential for the recovery of cartilage function (88). At the targeted repair areas, MSCs can release various cytokines, growth factors (GF), and chemokines, driving endogenous MSCs to enter lesion areas and creating an appropriate regenerative microenvironment while simultaneously aiding the regeneration of cartilage tissue (89). The combination of MSCs with exogenous biochemical or biomechanical stimuli, in addition to customized engineered scaffolds in MSC-based therapies, represents a significant advance in cartilage regeneration (89, 90).

Additionally, microfracture surgery is a commonly used technique for early-phase cartilage injury. In microfracture surgery, the surgeon drills several holes in the subchondral bone to discharge BMSCs, cytokines, and platelets from the marrow, which can stimulate the regeneration of cartilage (91, 92). Microfracture surgery is preferred by the majority of orthopedic surgeons for its simple single-stage technology and confined invasiveness (92). Furthermore, this approach was 90% successful in relieving pain postoperatively in cartilage lesions (93, 94). After performing microfracture surgery on full-thickness cartilage defects, histological evaluation of the early changes of the cartilage showed that the repair was induced by endochondral ossification in the depths of the microfracture punctures (95). Furthermore, endochondral ossification could activate osteoclasts and induce the reconstruction of cartilage, which regenerates earlier than subchondral bone. The Food and Drug Administration considers microfracture surgery to have a good prognosis in the treatment of small-sized cartilage injuries. Many types of research have shown that microfracture surgery can postpone cartilage degeneration, regardless of the lesion size (96, 97). However, some studies have reported that the post-surgical microenvironment of microfractures failed to induce the appropriate differentiation of BMSCs, leading to the formation of relatively unstable fibrous tissue rather than cartilage tissue (98).

Recently, in an attempt to identify an easy-to-handle cell substitute for MSCs, the stromal vascular fraction (SVF) was characterized for application in preclinical and clinical scenarios (95). The SVF includes not only ADSCs, but also a heterogeneous group of cells, such as progenitor cells, endothelial cells, fibroblasts, monocytes, macrophages, immune cells, muscle cells, pericytes, CD34^+^ cells, GFs, adipocytes, and stromal components (99).

Like MSCs, the SVF is proangiogenic and immunomodulatory, and its cellular components can differentiate and proliferate, all of which make it suitable for tissue regeneration (97). The advantage of using SVF over expanded ADSCs becomes immediately apparent because the SVF, obtained *via* digestion with collagenase and centrifugation of autologous adipose tissue, can be easily harvested by the patient themself through liposuction. It therefore requires minimal handling and contains ADSCs in a density ranging from 0.06 to 4 CFU-f. Thus, the SVF could be injected directly into damaged tissue, reducing inflammation, promoting regeneration, and resulting in reduced healthcare costs and fewer hours of hospitalization (99–101). Indeed, SVF allows for a “one-step” surgical procedure whereby the SVF can be harvested and implanted in the same surgical session, without requiring *in vitro* expansion (101, 102). This procedure involves minimal cell manipulation and low culture-related risks, with no specific regulatory requirements for clinical translation, thus expediting surgery. The process, from surgical harvesting of adipose tissue to the production of the SVFs and their seeding on a scaffold, hydrogel, or their direct injection, takes a maximum of 4 hours (101, 102).

The first reported example of successful sternal reconstruction using adipose-derived SVF stem cells was reported in 2015 by Zain Khalpey et al., in addition to traditional techniques (103). They used a 3D-printed model for setting the sternum and SVF, with the injection of 300 million cells both locally and intravenously, deposited at the level of the healed area of the sternum (103). The initial results were almost complete pain reduction and sternum nonunion after 6 months. Future studies will be needed to clarify the use of autologous stem cells from the SVF in combination with commonly used surgical approaches (103).

Several protein drugs exploit the fact that bone regeneration can also occur by stimulating tissue repair using GFs, which can regulate MSCs to restore the damaged tissues (104). Small molecules, compared to macromolecules, exert a major effect as they are less immunogenic and have higher osteoinductive potential, in addition to reduced manufacturing costs and contamination risks (105). These benefits have motivated the increasing number of studies regarding these molecular drugs in the last decade. However, there are some limitations to their clinical application: first, they are small enough to also penetrate non-specific cells and trigger undesirable signaling cascades; second, they have non-specific adverse effects; and third, they require an effective delivery strategy, which remains an issue as it is necessary to develop an engineered scaffold that modulates the appropriate amount of the drug (106, 107). More sophisticated studies and examples of drug delivery systems are required to overcome this limitation and support the use of these small drugs in regenerative medicine (108).

## Discussion

In addition to defining new approaches and techniques to reconstruct the chest wall and sternum, there is a need for each surgeon and to consider the most appropriate clinical course, which depends greatly on the clinic and material availability. The scientific community is pushing more and more frequently to use bioabsorbable materials, and the newest approaches are represented by computed tomography with reconstructed 3D images and the production of a 3D printed bioscaffold (67, 68).

These innovations may support different prostheses, tailored not only for the enhancement of the resection but also to adapt it to each patient.

Metcalfe and Ferguson suggested that the skin layers may eventually be replaced with biomaterials or stem cells, although the current ability to regenerate tissue is still too limited for large-scale surgery (109).

In particular, one of the most interesting and studied approach is the use of scaffoldless of neocartilage made by native tissue using expanded chondrocytes and various exogenous stimuli. Strategies have been set for the integration, although several techniques have been developed (109–111). Specifically, these approaches are set on the hurdles of cartilage regeneration, with particular attention on the fibroblast growth factor 18 (FGF-18) which induces cartilage growth and reduces cartilage degeneration in osteoarthritis (112, 113). New recent technologies are able to induce juvenile chondrocytes generation with MSCs (114) and scaffolds now include biphasic, osteochondral designs that may immediately bear load (115).

**Figure 4:**
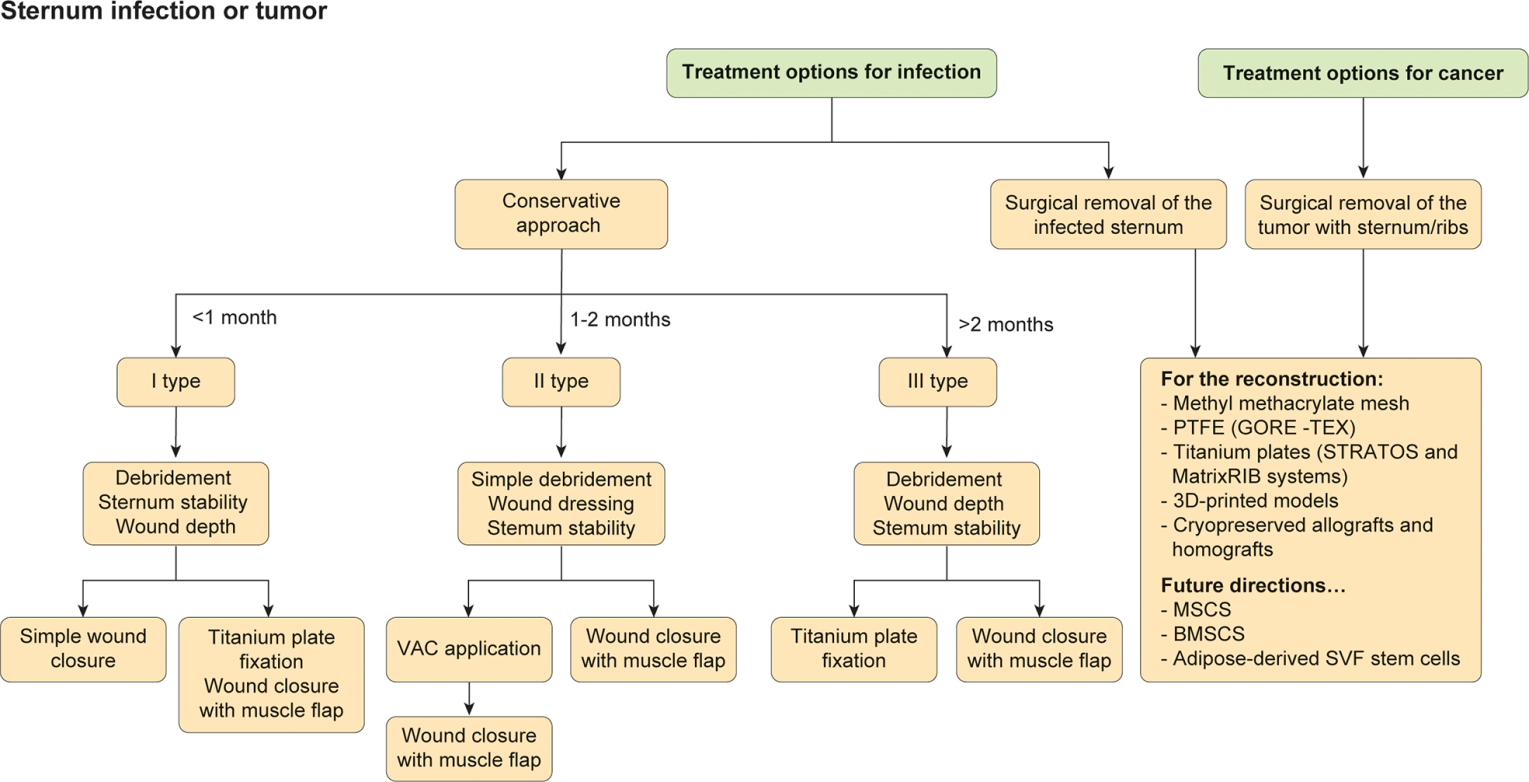
Treatments for sternal infections or tumor mass. The flowchart represents the different strategies in case of infection or cancer infiltrating the sternum.

The scaffoldless used also allow to the formation of constructs that can be immediately load-bearing upon implantation (36, 116). Another emerging approach is represented by the use of scaffolds with moieties, such as N-hydroxysuccinimide, that is able to bind collagen (117). The stimulation of the neocartilage by mechanical (28), anabolic (65), and, potentially, catabolic stimuli (65) may result in a synergistic interaction in cartilage formation. For FDA, new cartilage therapies should be resistant for long time. However, it is not well defined the calibration of the toughness and hardness, for the resistance to wear. In addition to mineralization, data on cartilage crosslinks in engineered or repair cartilages are not defined and described yet (118, 119). The next step of the use of new cartilage will be the durability test. However, though currently healing of cartilage defects continues to be elusive, given that emerging technologies are being validated clinically, the field is primed for an explosion of cartilage regeneration techniques that should excite those suffering from cartilage afflictions (118). Furthermore, while osteoarthritis is currently an intractable problem, exciting new discoveries bode well for the eventual healing of a problem that afflicts a quarter of our adult population. In conclusion, the most important aspect regarding chest wall defects is the severity of the lesion, including the condition, presence of infection, and presence or type of cancer. Additionally, the development and selection of appropriate biomaterials to reconstruct the thorax may improve the quality of life and long-term results. The choice to adopt one prosthesis instead of another one depends on the surgeon and specific clinic (**Figure 4**). Ultimately, a multidisciplinary team is necessary to assure more high-quality decisions.

## Author contributions

(I) Conception and design: BA and FS. (II) Administrative support: FS. (III) Provision of study materials or patients: BA and FS. (IV) Collection and assembly of data: BA and FS. (V) Data analysis and interpretation: BA and FS. (VI) Manuscript writing: BA, VM, LFZR; (VII) Revision end editing: MD, RS, FS. All authors contributed to the article and approved the submitted version.

## Acknowledgments

The Authors thank Prozetesis.org for the contribution.

## Conflict of interest

The authors declare that the research was conducted in the absence of any commercial or financial relationships that could be construed as a potential conflict of interest.

## Publisher’s note

All claims expressed in this article are solely those of the authors and do not necessarily represent those of their affiliated organizations, or those of the publisher, the editors and the reviewers. Any product that may be evaluated in this article, or claim that may be made by its manufacturer, is not guaranteed or endorsed by the publisher.
